# Blood Pressure Changes While Hiking at Moderate Altitudes: A Prospective Cohort Study

**DOI:** 10.3390/ijerph17217978

**Published:** 2020-10-30

**Authors:** Ky B. Stoltzfus, David Naylor, Tessa Cattermole, Arthur Ankeney, Rebecca Mount, Rong Chang, Cheryl A. Gibson

**Affiliations:** 1 Department of Internal Medicine, University of Kansas Medical Center, Kansas, KS 66160, USA; dnaylor@kumc.edu (D.N.); rmount2@kumc.edu (R.M.); cgibson@kumc.edu (C.A.G.); 2 Department of Surgery, University of Vermont Medical Center, Burlington, VT 05401, USA; tessa.cattermole@gmail.com; 3 Department of Emergency Medicine, University of South Carolina, Prisma Health Richland, Columbia, SC 29203, USA; a.ankeney@gmail.com; 4 Khoury College of Computer Sciences, Northeastern University, Boston, MA 02115, USA; changrongnj@gmail.com

**Keywords:** hypertension, hiking, recreation, altitude, cardiovascular, mountaineering

## Abstract

Recreational hiking in the mountains is a common activity, whether for a single day or for several days in a row. We sought to measure blood pressure (BP) response during a 10-day trek at moderate-altitude elevation (6500–13,000 feet) and observe for uncontrolled hypertension and/or adverse cardiovascular outcomes. A total of 1279 adult participants completed an observational study of resting BP during a 10-day trek in the Sangre de Cristo mountains. Following initial recruitment, participants were issued a trail data card to record BP measurements at day 0 (basecamp), day 3, day 6 and day 9. BP was measured using a sphygmomanometer and auscultation. Demographic data, height, weight, home altitude, daily water and sports drink intake, existence of pre-arrival hypertension and BP medication class were also recorded. We observed a rise in mean blood pressure for the cohort during all exposures to moderate altitudes. The increases were greatest for individuals with pre-existing hypertension and/or obesity. There were no observed life-threatening cardiovascular events for participants. We conclude that for individuals with a modestly controlled blood pressure of 160/95 mmHg, hiking at a moderate altitude is a safe activity.

## 1. Introduction

Hiking at elevated altitude is a popular outdoor activity in the United States and in many other countries. Hiking at moderate altitudes of 1500–2500 m (4900–8000 feet) over one–three weeks has been shown to provide positive health effects for individuals with metabolic syndrome and healthy individuals [1]. However, those with pre-existing hypertension have been found to be at increased risk of sudden death while taking ski vacations at high altitudes [2]. Studies of alpine hikers, skiers and mountaineering populations show that cardiovascular disease is prevalent, although perhaps at a lower rate than the general population [3,4]. A recent joint statement of clinical recommendations for high-altitude exposure of individuals with pre-existing cardiovascular conditions outlines class II-B evidence for checking BP before and during hiking activity for individuals with moderate-severe hypertension [5]. Furthermore, it cites class I-C evidence for the safety of individuals with well-controlled hypertension to reach high altitude (>13,000 feet) and I-C evidence that uncontrolled hypertensive individuals avoid high-altitude exposure due to the risk of organ damage. 

The physiologic mechanisms of elevation in BP with altitude are complex and dynamic, influenced by factors such as time spent at altitude and altitude level [6]. Study of normotensive individuals has shown mixed results of decreased BP, increased BP and no change in BP with exposure to moderate or high altitude [7,8,9]. Using the altitude classification of previous review articles of moderate altitude at 4900–8000 feet and high altitude at 4900–11,500 feet [5,10], there are few studies that address BP changes for moderate to high altitudes in the general population. A recently published cohort study of 672 Himalayan trekkers by Keyes et al. showed that exposure to altitudes of 2860–4300 meters (9400–14,100 feet) resulted in no significant change in BP for normotensive or hypertensive participants [9]. However, the prevalence of pre-existing hypertension in this study was low (only 60 patients) and limitations in data collection precluded reporting of home baseline BPs. Providing medical advice to individuals hiking at moderate to high altitude remains unclear [11]. 

Philmont Scout Ranch (PSR) is operated by the Boy Scouts of America in Cimarron, New Mexico, USA. The ranch is 567 km^2^ and has about 23,000 youth and adults who participate in a summer hiking trek each year. A group of clinicians providing medical oversight to the infirmary operations at PSR asked which participants may be at greatest risk for developing hypertension at moderate to high altitude. Furthermore, PSR has a current policy of restricting individuals with BP >160/95 mmHg from hiking until BP is better controlled. This policy has been questioned as to its clinical veracity in preventing serious health consequences for participants at PSR. The objective of this observational cohort study was to measure BP response for normotensive and hypertensive individuals during a 10-day hiking trek at moderate-altitude elevation (6500–13,000 feet) to observe whether participants had a clinically significant rise in blood pressure or if any participants had an acute adverse cardiovascular health event such as acute coronary syndrome, hypertensive crisis or acute stroke. These data were intended to determine whether the current guideline used at PSR of allowing participants to hike if his/her blood pressure is <160/95 is effective in safely preventing adverse health outcomes.

## 2. Materials and Methods

### 2.1. Study Participants

Study participants were recruited from Philmont Scout Ranch (PSR), located in Cimarron, New Mexico, USA, during two separate summer seasons (May–August, 2015, and May–August, 2016). Prior to arrival, all PSR visitors were required to have a screening history and physical exam performed by their respective primary care physicians (PCP) to verify that he/she was in good health and record vital signs in their home environment. All adult PSR hikers, aged 18 years or older, who were deemed in good health by their physicians, were eligible to participate in the study. Study exclusion criteria included exceeding maximum weight for height limit restrictions (e.g., at a height of 72 inches, a person cannot weigh more than 239 pounds) and having a pre-existing condition that would preclude hiking more than 90 miles over a 10-day period as determined by a PSR staff physician on a case-by-case basis (e.g., uncontrolled anginal chest pain). Participants reporting pre-existing hypertension must have been stable on medication(s) with controlled BP less than 160/95 mmHg. Additionally, all study participants were required to refrain from alcohol consumption and tobacco use during their trek (neither product is provided in meal bags by PSR or available for purchase at commissary stops). 

Participants were acclimatized to an altitude of 6702 feet at the PSR basecamp for 2 days prior to hiking. Recommendations were provided to all participants to hike moderate to long distances (4–10 miles) at their home altitude for 2–3 months prior to arrival at PSR. During the 10-day trek, participants were exposed to altitudes of 6702–12,440 feet while hiking. However, BPs were measured at camps where the participants stopped for a prolonged break (2 h or greater) or spent the night, ranging from altitudes of 6946–10,532 feet. 

The Institutional Review Board at the University of Kansas Medical Center approved the study protocol. All participants gave verbal consent prior to enrollment in the study.

### 2.2. Health History and Medication Use

Clinical data were extracted from the participant’s health information record, which was completed by the participant prior to arrival at PSR and reviewed by his/her PCP. Information included baseline demographic and health data, including age and gender, height, weight, body mass index (BMI), home BP and pulse. Health history, which was self-reported and verified by the participants’ primary care physician, included pre-existing health conditions and current medication(s) with dose and frequency. 

### 2.3. Trail Data Measurement

Each participant carried a trail data card throughout his/her trek and data were recorded for the participant. The participant returned the trail data card to the research coordinator upon completion of his/her trek. The trail data card included the location of BP measurement, time of day BP was taken, water intake for the 24-h period prior to BP recording and sports drink intake for the 24-h period prior to BP recording. Participants were instructed not to fill in missing data or record BP values that were not taken by PSR staff.

### 2.4. Blood Pressure Measurement

All participants were encouraged to maximize oral hydration prior to and during their hiking trek. Participants were instructed to monitor daily urine output as a measure of hydration with the general guidance that “urine clear in color and copious in amount” indicated adequate hydration. A participant would consume a rehydrated sports drink mix (high sodium content) at his/her discretion to aid in hydration.

BP was measured using the conventional auscultation method by PSR staff. Participants were encouraged to have BP measured during the morning hours, however, due to constraints of beginning hikes at early hours (6:00 AM or earlier), BP was sometimes measured later in the day for the convenience of the participant. We had no mechanism available to enforce the time of day that BP would be measured. Ranch staff were trained on proper BP measurement. The research coordinator made random quality control checks throughout the study period to ensure ranch staff obtained BP measurements in an accurate and precise manner. Participants were instructed to have their BP measured in the afternoon or evening after completing hiking activity for the day. Data were recorded for the actual time of day that BP was obtained.

Arm circumference was measured on the dominant arm to determine the appropriate cuff size (regular or large). Before the initial BP measurement, participants were instructed to rest for 5 min while seated. After the initial measurement, the participant rested for an additional 5 min before the second BP measurement was taken. Both values were recorded on the participant’s study card by the ranch staff. The average of the two BP readings was calculated. Research personnel decided a priori to measure BP on days 0, 3, 6 and 9 as a convenience sample.

BP measurements were taken on days 0, 3, 6 and 9 of the 10-day trek with the corresponding altitude at which the BP was taken. The day 0 measurement was always performed at basecamp. Home BP was obtained from the participant’s health form (as recorded by the participant’s primary care physician). Measurements on days 3, 6 and 9 were performed at various altitudes, according to the trek itinerary of the participant. Participants may have reached higher altitudes while hiking on these days. Participants followed unique itineraries across the ranch and were at different camp locations of differing altitudes on days 3, 6 and 9.

Participants who returned their trail data card were included in final analysis. 

### 2.5. Statistics

Descriptive and multivariate analyses were conducted. A descriptive analysis was conducted to estimate the mean and standard deviation (SD) for continuous variables, and frequencies and proportions for categorical variables. A series of *t* tests and regression analyses was then performed to further determine how blood pressure varies with the associated effects of our hypothesized predictors (altitude, prior health conditions and demographic data). The Pearson’s correlation test was used to compare continuous variables. To compare differences between categorical groups, ANOVA tests and Student’s t-tests were used. Co-variates with statistical and clinical significance were entered into the multivariate linear regression model. All tests conducted were two-tailed tests. To evaluate statistical significance, *p*-values were reported. All analyses were conducted using IBM SPSS Statistics for Windows, Version 23.0 (IBM Corp., Armonk, NY, USA).

In addition to the main effect of altitude elevation, we hypothesized individuals’ characteristics may have associated effects on the changes of blood pressure among our participants. We performed a series of *t* tests (i.e., the test to measure the difference between groups) to determine whether these effects were significant to be further incorporated in our regression analysis, which explored the effects of altitude and time on the change of blood pressure. For this comparison purpose, the continuous variables of age and BMI were coded as binary indicators. Specifically, age was categorized into the old group (age at and over 50) and the young group (age between 20 and 49), as the mean of age in the population was 49. BMI was categorized as the normal group (BMI less than 25) and the overweight group (BMI equal and over 25).

In this study, all the participants experienced the time and altitude changes over the trek. A linear regression analysis is appropriate for us to determine the influence of altitude elevation on blood pressure across the four measurement times, which could be represented as

*Blood Pressure (BP) = individual variation of BP + time effect + altitude effect + interaction effect of altitude × time + measurement error*

**Figure 1:**
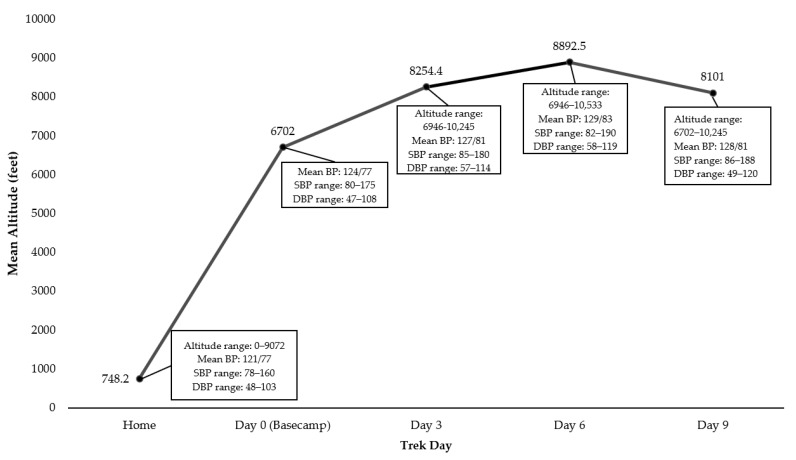
Timeline, altitude and blood pressure.

In addition, we observed that the changes of blood pressure for each participant were not consistent over the trek (Figure 1). The variation in the change pattern of blood pressure was due to individual differences. Thus, the individual factors of gender, BMI and hypertension history that we found significant through *t* tests were included in our regression model for better interpretation. These factors could be represented as follows:

*Individual variation of BP = gender difference + BMI difference + hypertension history + measurement error*

## 3. Results

### 3.1. Participants

Overall, 1279 of 2008 participants completed the study during the summer camping seasons of 2015 and 2016 (see Table 1). Individuals who did not complete the study failed to return their trail data cards. Our study population was overwhelmingly male and with ages predominantly in the 40–59 years old range. The mean age of participants was 49 (*SD* = 7.5) years, with the youngest participant at the age of 20 and the oldest at the age of 72. For both cohorts, most participants were between the ages of 40 and 59 years. The BMI of most participants was in the overweight range (52.8%) or obese range (24.6%). A minority of patients had a self-reported prior history of hypertension (9.5%). 

### 3.2. Blood Pressure

The mean home blood pressure measurements indicated normotensive readings (120.9/76.9 mmHg ± 10.9/7.7). Figure 1 shows changes in mean BP measurements for all participants at days 0, 3, 6 and 9. The mean of blood pressure increased through day 6 (*M*_day3_ = 127.3/81.5 mmHg ± 11.3/8.5; *M*_day6_ = 129.1/82.6 mmHg ± 11.0/7.9) and then decreased at day 9 (127.7/80.8 mmHg ± 10.7/8.2). Blood pressure was influenced by both altitude and the time at higher altitudes.

Time demonstrated a significant fixed effect on the change of SBP (γ = 0.42, *p* < 0.05), meaning that participants’ SBP increased on average by 0.42 mmHg (*SE* = 0.36 mmHg) for every three days during the trek. The significant random effect of time (µ = 5.21, *p* < 0.001) showed that such changes of SBP due to the time were significantly different between time periods, and that the amount of changes in SBP at the earlier time was smaller than at the later time. As expected, the evaluation of altitude demonstrated a very strong and positive effect on the change of SBP (γ = 0.0016, *p* < 0.001). That is, for every unit (i.e., feet) elevation in altitude, SBP increased about 0.0016 mmHg, on average. The negative parameter of interaction effects from the altitude across the measurement time (γ = -0.0005, *p* = 0.165) revealed that with the evaluation of altitudes raised across the time, the amount of increase in SBP became smaller than at the lower altitude, yet this difference was relatively small and not significant. 

All the personal condition variables showed significant fixed effects on SBP. Older participants showed higher SBP (γ = 0.09, *p* < 0.005), and this is consistent during the change of altitudes over time, as revealed by the group comparison results. Males had significantly higher SBP than females at different altitudes (γ = 4.27, *p* < 0.001). BMI was another significant factor on the increase in SBP (γ = 0.67, *p* < 0.001). Moreover, history of hypertension also demonstrated strong statistical effects on SBP (γ = 2.75, *p* < 0.001), indicating participants with a history of hypertension had higher SBP over time. The intraclass correlation coefficient (ICC) of the regression model was 0.560, suggesting about 56% of the variance in SBP over the time could be explained by these four personal conditions (i.e., age, gender, BMI, history of hypertension). 

Time did not show a significant effect (γ = 0.18, *p* = 0.115) on the change of DBP. Specifically, every unit of measurement time change led to an average of 0.18 mmHg (*SE* = 0.161 mmHg) increase in DBP, but this effect was not significant. Altitude demonstrated a significant and strong fixed effect (γ = 0.0025, *p* < 0.001), meaning that for every unit change in altitude, this resulted in an average 0.0025 mmHg change in DBP. A significant interaction effect of altitude over time was also found, with the increase in DBP due to altitude becoming less at the higher altitude level. Consistent with *t* test results, age was negatively related to the changes of DBP over time. Younger participants showed relatively higher DBP than older peers over the elevation of the altitude. Besides age, other personal condition variables (i.e., gender, BMI and history of hypertension) showed similar significant and positive effects on the change of DBP. The final ICC revealed that about 34% of the variance in DBP over time could be explained by the personal conditions. 

### 3.3. Altitude

The initial altitude of every participant measured at basecamp on day 0 was 6702 feet (Figure 1). The mean altitude of participants’ home residence was 748.2 feet (*SD* = 1,114.4), averaging about 5954 feet below the altitude of basecamp. All the participants were exposed to various moderate to high altitudes during the next 10 days of the trek. Altitudes over the trek were associated with different time points. The mean altitude experienced by participants at each measurement time point varied, with a gradual altitude increase through day 6 (*M*_day3_ = 8,254.4 feet ± 779.3; *M*_day6_ = 8,892.5 feet ± 871.5), followed by a slight decrease at day 9 (8,101.0 feet ± 746.6). 

### 3.4. Group Differences by Participants’ Characteristics

As shown in Table 2, men had higher levels of blood pressure readings than women. However, differences of SBP and DBP between men and women were larger at the initial measurement time (day 0), *p* < 0.001, and gradually equalized with time and changes of altitude. 

The differences due to age were not consistent for SBP and DBP. Younger participants tended to have lower SBP than older individuals, yet such differences became smaller with the increase in mean altitude. Paradoxically, older participants showed slightly lower DBP than younger ones and the difference became significant with the increase in mean altitude. Overweight/obese participants consistently demonstrated significantly higher SBP and DBP than their normal weight peers throughout the trek. Participants with a history of hypertension also demonstrated statistically higher blood pressure compared to ones with no prior history.

Overall, the differences of blood pressure due to the factors of gender, BMI and hypertension history were obvious. These differences, however, became smaller over time during the trek.

### 3.5. Serious Medical Events

Urgent or emergent medical events related to hypertension were not reported for any participants. None of the participants were referred for treatment at the ranch infirmary for a cardiovascular event (myocardial infarction, hypertensive crisis and stroke). None were referred for further treatment of accelerated hypertension (defined by BP > 160/95 mmHg) once their trek had begun. None had symptoms prompting evaluation for accelerated hypertension (defined as blurred vision, shortness of breath, severe anxiety or chest pain). The maximum BP for any individual during any measurement day of their hiking trek was SBP 190 mmHg and DBP 111 mmHg.

### 3.6. Multivariate Analysis

History of hypertension and male gender had the greatest effect on the SBP of normotensive versus hypertensive participants. Time (as represented by day of trek), altitude, age, gender and BMI also had an effect on SBP but to a lesser degree. Similarly, history of hypertension and male gender had the greatest effects on DBP. However, only altitude and BMI had lesser effects. This is represented in Table 3.

## 4. Discussion

In this relatively large prospective cohort study, we show that for most individuals hiking at moderate altitudes, resting BP does not rise in a clinically significant manner for an individual. We did not measure active, or ambulatory, blood pressures in this study. None of the participants in our study suffered a life-threatening effect of elevated blood pressure. Participants who are obese and/or with pre-existing hypertension had a greater rise in their blood pressure and some clinicians may find this concerning. The findings our of study support the current pre-trail screening efforts for our participants. The initial rise in BP followed by a gradual rise as altitude increased has been well described in other papers and is validated here.

This information may also help guide healthcare providers in anticipating BP changes when travel to moderate altitudes is planned. Further study of this population may answer questions such as optimal pre-arrival medication guidance to mitigate BP response in those at higher risk of clinically significant events, specifically in ACE inhibitor use. Previous studies have examined high-altitude exposure and cardiovascular risks [12], but to our knowledge, there are no studies examining cardiovascular risk for the middle-aged, overweight or obese casual hiker.

For camps or other moderate-altitude trekking operations, this study may influence policy and procedures for the monitoring and management of hypertension. Philmont Scout Ranch is a high adventure camping and trekking operation with a large participant population of primarily adolescent and middle-aged people, but of diverse geographic home communities. Safety is of primary concern to the operations of the ranch with extensive pre-trail health screening. Potential health risks, including development of altitude-related hypertension, affect policy for whether adult leaders may participate in a trek. We support pre-trail screening and pre-arrival mitigation in HTN and other risk groups, when possible. In addition, further study identifying population groups that are at risk for higher than normal elevations in BP with altitude exposure is warranted.

### Strengths and Limitations

A major strength of this study was that it was conducted in a real-world environment, utilizing existing camp staff and infrastructure. As such, our study findings provide needed information about recreational hiking treks and the risk of health issues that can be used to guide medical advice to individuals hiking at moderate to high altitudes. Another strength of our study was that it included a large sample, enhancing the generalizability of findings. 

One of the limitations of this study was that the study population was primarily middle-aged men and race and ethnicity were not directly collected from participants. However, the Boy Scouts of America guides the PSR’s inclusion policy, which is non-discriminatory. PSR hikers include all ethnicities, religions and gender orientations. Another limitation was that study participants were individuals who were considered to be in good enough health to participate in a 10-day hiking trip prior to their arrival at the camp, which might have excluded some individuals with chronic health problems and potentially those with uncontrolled hypertension. Further, study participants were instructed to ideally have their blood pressure measured in the morning before they began hiking. However, this was not always feasible due to their need to begin hiking at early hours before camp staff were available to check blood pressure. Consequently, the time of blood pressure measurements was not consistent. Although we strongly recommended blood pressure measurements to be taken in the morning hours, practical constraints limited strict enforcement. To control for this factor, we required training staff to have the participant undergo two blood pressure measurements with rest required before the initial measurement.

## 5. Conclusions

Moderate-altitude exposure during hiking treks may not pose a significant risk for physically fit adults. There are differences observed for individuals with pre-existing hypertension and obesity and further study is warranted to clarify the degree and dose-dependent nature of these effects. However, our study confirms that most adults with controlled medical problems should be able to safely hike at moderate altitudes without consequent deleterious health problems.

## Figures and Tables

**Table 1:** Participant characteristics.

Characteristic	Total Sample (*n* = 1279)
Female	96 (7.5%)
Male	1183 (92.5%)
Age	49.0 (7.5)
Age 20–29	31 (2.4%)
Age 30–39	47 (3.7%)
Age 40–49	611 (47.8%)
Age 50–59	498 (38.9%)
Age 60–69	85 (6.6%)
Age 70 and older	7 (0.5%)
Altitude of residence, feet	748.2 (SD +/−1114.4)
Body mass index (BMI)	27.5 (3.2)
Underweight (BMI 15–19)	14 (1.1%)
Normal weight (BMI 2–24)	274 (21.4%)
Overweight (BMI 25–29)	675 (52.8%)
Obese (BMI ≥30)	315 (24.6%)
History of hypertension	208 (9.5%)
Blood pressure at home (mmHg)	120.9/76.9 (SD +/− 10.9/7.9)

**Table 2:** Group difference by level-2 predictors.

Characteristic	SBP			DBP		
	Mean of Group 1	Mean of Group 2	*T*	Mean of Group 1	Mean of Group 2	*t*
Gender	Male	Female		Male	Female	
Day 0	125.2	114.4	7.7 ***	77.7	71.9	6.4 ***
Day 3	127.8	121.5	4.6 ***	81.7	79.3	2.3 *
Day 6	129.4	124.7	3.8 ***	82.8	79.9	3.2 **
Day 9	128.0	124.4	2.6 *	80.9	79.3	1.7
Age	≥50 years	<50 years		≥50 years	<50 years	
Day 0	125.9	123.2	3.6 ***	77.2	77.3	−0.1
Day 3	128.0	126.8	1.9	80.8	82.1	−2.7 *
Day 6	130.1	128.2	2.9 **	82.0	83.1	−2.4 *
Day 9	128.5	127.0	2.5 *	79.9	81.6	−3.5 ***
BMI	≥25	<25		≥25	<25	
Day 0	125.9	119.3	7.6 ***	78.1	74.3	7.0 ***
Day 3	128.3	123.8	5.9 ***	82.1	79.4	4.6 ***
Day 6	129.8	126.6	4.5 ***	83.1	80.8	4.3 ***
Day 9	128.4	125.2	4.6 ***	81.2	79.4	3.4 ***
Hypertension History	Yes	No		Yes	No	
Day 0	130.9	123.2	7.5 ***	80.2	76.7	5.8 ***
Day 3	129.9	126.8	3.4 ***	82.7	81.2	2.2 *
Day 6	131.9	128.5	3.9 ***	84.1	82.3	2.7 *
Day 9	130.8	127.1	4.4 ***	82.1	80.5	2.1 *

Note. Statistical significance level: * *p* < 0.05, ** *p* < 0.01, *** *p* < 0.001.

**Table 3:** Estimates from the multilevel analysis of the change in systolic blood pressure over time.

Category	SBP		DBP	
**Fixed Effect**	**Estimate**	**SE**	**Estimate**	**SE**
Intercept	126.34 ***	0.36	80.11 ***	0.25
Time	0.42 *	0.15	0.18	0.12
Altitude	0.0016 ***	0.0002	0.0025 ***	0.0002
Altitude × Time	−0.0002	0.0001	−0.0007 ***	0.0001
Age	0.09 **	0.03	−0.07 ***	0.02
Gender (Male)	4.27 ***	0.90	2.36 ***	0.60
BMI	0.67 ***	0.07	0.42 ***	0.05
Hypertension history	2.75 ***	0.65	1.74 ***	0.44
**Random Effect**	**Estimate**	**SD**	**Estimate**	**SD**
Intercept	80.88	8.99	22.89	4.79
Time	5.21	2.28	1.10	1.05

Note: ICC^SBP^ = 0.560; ICC^DBP^ = 0.344. Statistical significance level: * *p* < 0.05, ** *p* < 0.005, *** *p* < 0.001.
